# Superoxide Scavenging Effects of Some Novel Bis-Ligands and Their Solvated Metal Complexes Prepared by the Reaction of Ligands with Aluminum, Copper and Lanthanum Ions

**DOI:** 10.3390/molecules18066128

**Published:** 2013-05-23

**Authors:** Shigeki Kobayashi, Sachi Kanai

**Affiliations:** Division of Analytical Chemistry of Medicines, Showa Pharmaceutical University, 3-3165 Higashitamagawagakuen, Machida, Tokyo 194-8543, Japan; E-Mail: dog-rackykun6.santafe@docomo.ne.jp

**Keywords:** superoxide, catechol, *p*-hydroquinone, antioxidants, density functional theory, aluminum, copper, lanthanum, solvated metal complexes

## Abstract

Novel antioxidants have been synthesized and characterized by their chemical properties as antioxidants with high superoxide scavenging activity. (2*R*,3*R*)-diphenylethylenediamine is a spacer in antioxidants, and we synthesized targets **11a** and **11b** by conjugation with *o*-hydroquinone and *p*-hydroquinone at the two N-termini, respectively. Superoxide scavenging activities of the plant flavonoid-like **11a** and **11b** were compared with those of known antioxidants, and shown to increase in the following order: curcumin << ascorbic acid < Trolox < (+)-epicatechin < **11a** < quercetin ° **11b**. Compound **11a** also formed a solvated **11a**–metal complex with metal ions. The **11a**–Cu^2+^ complex was shown to have higher superoxide scavenging activity than that of **11a**, **11b**, Cu^2+^, and the **11a**–Al^3+^ and **11a**–La^3+^ complexes, whereas the **11a**–Al^3+^ complex increased rather than decreased superoxide levels. The **11a**–Al^3+^ complex did not abstract one electron from the SOMO of O_2_**^−.^** since the HOMO and LUMO phases of Al^3+^ do not exist in the center of the **11a**–Al^3+^ complex. However, the SOMO of the **11a**–Cu^2+^ complex distributed in the center of Cu^2+^ may abstract one electron from the SOMO of O_2_**^−.^**. These results suggest that **11a** and **11b** are powerful antioxidants.

## 1. Introduction

Superoxide (O_2_^−**.**^) and the hydroxyl radical (OH**^.^**) are produced by the reduction of O_2_ and have doublet spin multiplicity. These radicals are highly reactive and cause oxidative damage by the abstraction of an electron or H radical from biomolecules, especially proteins, lipids, and DNA. The excessive production of reactive oxygen species (ROS) in the body may be the most dangerous factor of many diseases such as inflammation, diabetes, cancer, aging, and neurodegenerative diseases including Parkinson’s and Alzheimer’s diseases [1,2]. Therefore, in recent years, many studies have investigated how to protect against ROS-induced oxidative damage using natural antioxidants such as vitamin E, vitamin C, and plant products such as flavonoids [3,4,5]. Russo *et al.* compared radical scavenging activities using a correlation between O-H bond dissociation enthalpy (BDE) and the ionization potential (Ip) for the mechanism of the free radical oxidation of flavonoids [6,7]. Reactions between the antioxidants and metal complexes of flavonoids and OH or OOH radicals have also been investigated using computational kinetics methods [8]. Moreover, the metal ion effect for the radical scavenging of catechin has been investigated by kinetics analyses using the stopped flow method [9]. However, the design and production of more effective synthesized antioxidants and antioxidative metal complexes with their related products are less challenging than those of natural antioxidants. 

The electronic states and estimation of the scavenging activities of antioxidants [10,11], using absolute hardness (η) and electronegativity (χ) based on the chemical hardness theory [12,13,14], are very important to consider for the design process of antioxidants. We paid particular attention to the catechol ring substituted at C2 of the B ring in flavonoids because our previous studies have shown that the catechol ring produces chemically soft flavonoids. Conjugation of the catechol ring increased the electron donation, η, and χ of flavonoids [10]. *p*-Hydroquinone is a likely catechol ring with a similar radical scavenging activity to catechol. In this study, the radical scavenging activities of natural products and the novel antioxidants, **11a** and **11b**, were estimated by a reaction with O_2_^−**.**^, which was yielded from the hypoxanthine (HPX)-xanthine oxidase (XOD) system. We showed that the O_2_^−**.**^ scavenging activity of **11a** and **11b** was higher than that of ascorbic acid, (+)-epicatechin, curcumin, Trolox, catechol, and *p*-hydroquinone.

We previously reported the synthesis and chemical properties of the solved complex of bis(amino acid)catechol derivatives with lanthanoid (Ln^3+^) ions [11,15,16]. We paid attention to the fact that Al^3+^ and Cu^2+/+^ ions, besides amyloid β protein misfolding and self-assembly, are causative compounds of Alzheimer’s disease [17,18]. To investigate the influence of metal ions and metal complexes on the production and elimination of ROS, we measured O_2_**^−.^** scavenging ability through chelation since **11a** could form metal complexes with Al^3+^ and Cu^2+^ ions. It has been suggested as the active site since the **11a**–Al^3+^ and **11a**–La^3+^ complexes have no or less effective O_2_**^−.^** scavenging activity. However, we found that low concentrations of the Al^3+^ ion slightly accelerated the formation of O_2_^−**.**^ by binding with **11a**.

To anticipate the strength of potency for the O_2_^−**.**^ scavenging activities of Al^3+^, Cu^2+^, and La^3+^, we discussed the electronic states of Al^3+^, Cu^2+^, La^3+^, **11a**–Al, **11a**–Cu, and **11a**–La complexes using the chemical hardness theory described above. The absolute hardness of Cu^2+^ is smaller than that of the Al^3+^ and La^3+^ ions since the coordinates **r**(χ, η) as the electronic structures of Al^3+^, Cu^2+^, and La^3+^ ions have been shown to be **r**(70.48, 37.96), **r**(25.55, 4.7), and **r**(15.23, 14.43), respectively, in a ground state [10,11]. A singly occupied molecular orbital (SOMO) of the *d* orbital of **11a**–Cu^2+^ is widely distributed in the center of the metal ion. The **11a**–Cu^2+^ complex easily abstracted an electron from O_2_^−**.**^; therefore, we demonstrated that **11a**, **11b**, **12**, **13** and the **11a**–Cu^2+^ complex are useful antioxidants. 

## 2. Results and Discussion

### 2.1. Antioxidant Activity of Several Natural Producuts

**Figure 1:**
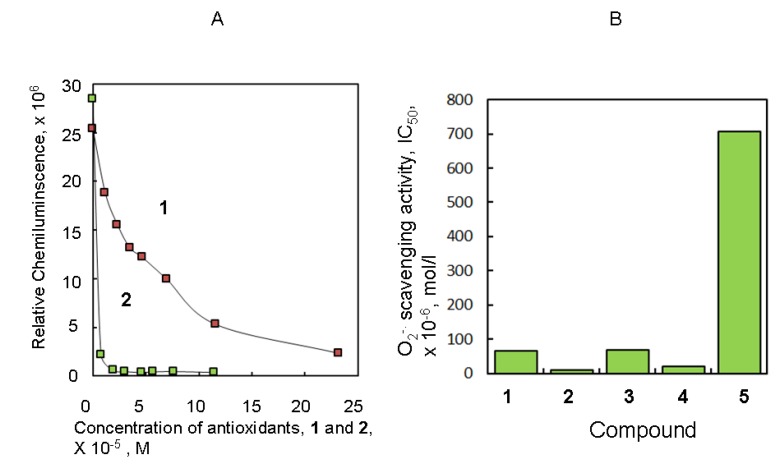
Dose–response curve of Trolox (**1**) and quercetin (**2**) on O_2_^−**.**^ scavenging inhibition (**A**) and the IC_50_ values of several antioxidants (**B**). All bars were expressed as the mean.

O_2_^−**.**^ scavenging activity was determined from the half maximal inhibitory concentration (IC_50_) by changes in the chemiluminescence (CL) response by the reaction of 2-methyl-6-*p*-methoxyphenyl ethynylimidazopyrazynone (MPEC) with the O_2_^−**.**^ produced from the HPX-XOD system [19]. The IC_50_ represents the 50% concentration point of the dose-response curve of CL responses with various concentrations of antioxidants. Figure 1A shows the dose-response curve for O_2_^−**.**^ scavenging measured at 7–8 points of a concentration range between 0.0 and 10^−4^ M (mol/L) of Trolox (**1**) and quercetin (**2**). Trolox is a well-known antioxidant model of *dl*-α-tocopherol (vitamin E) [20]. In this experiment, relative CL intensity was about 20–30 × 10^6^ relative chemiluminescence units (RLU). The O_2_^−**.**^ scavenging activities of **1** and **2** were 64.7 and 8.8 µM. The activities of L-ascorbic acid (**3**), curcumin (**5**), and (+)-epicatechin (**4**) were 68.8, 708, and 21.1 µM, respectively, from the results obtained using the same experimental method. These results are summarized in Figure 1B. Antioxidative activities were shown to increase in the following order: **5** < **3** < **1** < **4** < **2** (Figure 1). 

One of the reasons why curcumin has lower O_2_^−**.**^ scavenging activity than epicatechin is because the catecholic hydroxyl of curcumin protects the CH_3_ group. In the compounds tested in this study, the activity of quercetin was the most potent. The reason for this was the activity of the catechol ring. The catechol ring naturally binds at C2 in flavonoids such as quercetin, cyanidine, and rutin. Although flavonoids are expected to be natural products that provide powerful radical-scavenging activity, natural flavonoids in which the *p*-hydroquinone ring, a well-known regioisomer of catechol, binds to the flavonoid ring are almost unknown. The catechol and *p*-hydroquinone rings are useful in the development of new potent antioxidants. 

### 2.2. Synthesis and Stable Conformation of ***11a*** and ***11b***

**Scheme 1:**
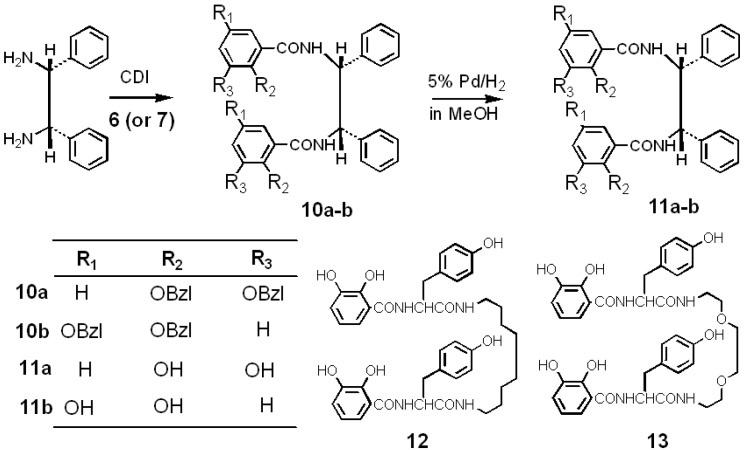
Synthesis of the new antioxidants **11a** and **11b**.

Interestingly, quercetin and epicatechin substituting catechol to the B ring of flavone and flavanone increased O_2_^−**.**^ scavenging activity and we designed targets **11a** and **11b** conjugated with two 2,3-bis(benzyloxy)benzoic acid (**6**) and 2,5-bis(benzyloxy)benzoic acid (**7**) units at the N-terminus of the (1*R*,2*R*)-(−)-1, 2-diphenylethylenediamine spacer, respectively, as shown in Scheme 1.

In particular, 2,5-bis(benzyloxy)benzoic acid was comparable to *p*-hydroquinone (**8**). Compound **11a**, a derivative of catechol (**9**), was expected in the formation of a metal complex with metal ions [20,21]. Yields of **11a** and **11b** were lower than those of **12** and **13** due to the steric inhibition of 1,2-diphenyl groups. The crude compounds **10a** and **10b** were purified using column chromatography over neutral silica gel, respectively, and were recrystallized from methanol. After the purification of **10a** and **10b**, the benzyloxy protecting groups were removed with H_2_ and 5% Pd-C in methanol to yield the targets **11a** (50%) and **11b** (55%), respectively. Here, for example, compound **11a** was purified by preparative thin layer chromatography (PTLC) using a silica gel plate with chloroform/MeOH (20:1) as mobile phase. Compounds **12** and **13** were synthesized following the same method as for **11a** and **11b** [10,11,12]. The compounds **11a**, **11b**, **12**, and **13** provided satisfactory analysis by ^1^H-NMR, ^13^C-NMR, ^13^C-^1^H COSY NMR, and FAB-MS. The tyrosine (Y) α protons of **12** and **13** observed at δ 4.70 and 4.74 are coupled with an amido proton at δ 8.49 and 8.46, respectively, and the observed ^3^J_α,NH_ values are 7.1–7.8 Hz. The values indicate that **12** and **13** provide a folded β-sheet like conformation [21]. Moreover, a very broad amido proton observed at δ 9.0 may be consistent with an intramolecular hydrogen-bonded structure in double-strands of **13**. On the other hand, **11a** and **11b** are assumed to have twist conformations since the half width values of J_3H,NH_ of their amido protons were <3 Hz.

**Figure 2:**
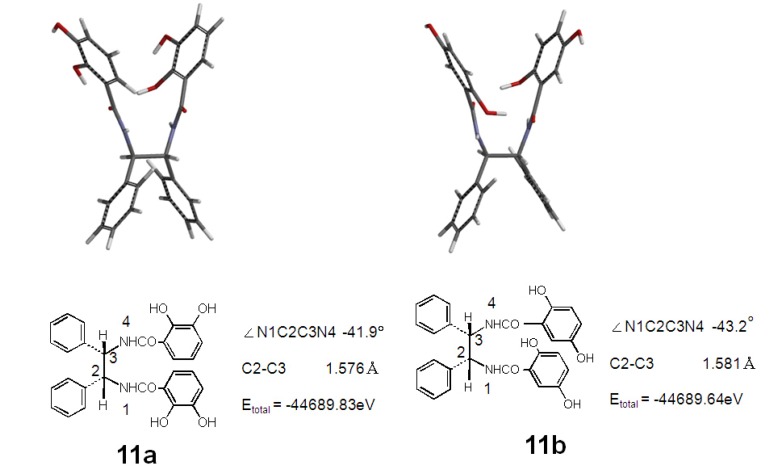
Most stable conformations after geometry optimization of **11a** and **11b**.

**Figure 3:**
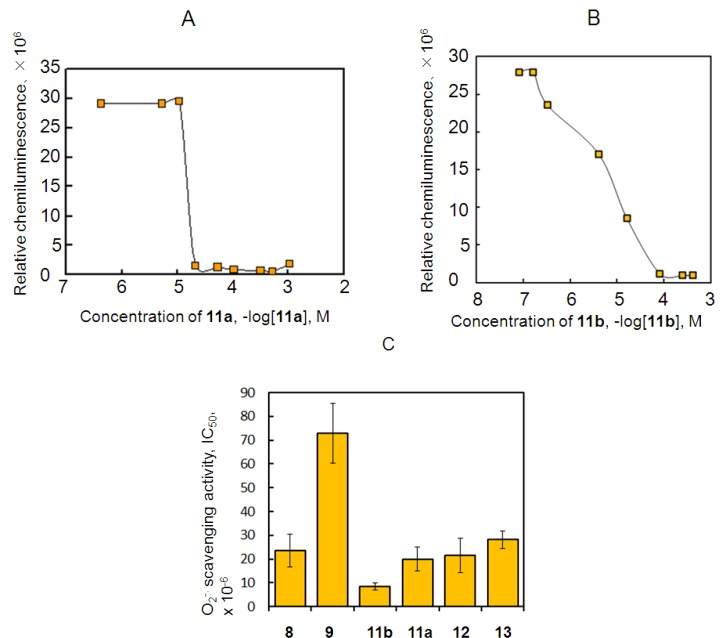
Dose–response curve of the synthesized antioxidants **11a** (**A**) and **11b** (**B**) on O_2_^−**.**^ scavenging activity and the IC_50_ values of several antioxidants (**C**).

Compounds **11a** and **11b** had two catechol or *p*-hydroquinone rings in one molecule, respectively. When the O_2_^−**.**^ scavenging activities of **11a** and **11b** were compared with those of the catechol and *p*-hydroquinone rings, the potency of their activities was different. Compounds **11a** and **11b** had powerful antioxidant activity similar to quercetin and (+)-epicatechin. Hence, we estimated the conformation of **11a** and **11b** using a computational method by density functional theory (DFT). The most stable conformations of **11a** and **11b** were computed using conformation analysis with the MMFF method. Details of the conformation and electronic state were obtained after geometry optimization using B3LYP with a 6-31G(d) basis set and the B3LYP/6-31G(d) computed results are shown in Figure 3. The most stable structures of **11a** and **11b** in the gas phase are also shown in Figure 2. Total electron energy (E) was more stable in compound **11a** (−44689.83 eV) than in **11b** (−44689.64 eV). The two catechol rings of **11a** faced each other. From this conformation, it is suggested that the four hydroxyl groups of **11a** can easily form the metal chelator (Figure 2).

At B3LYP/6-31G(d) level; ∠N1C2C3N4 is the dihedral angle (°), C2-C3 is bond length (Å), and E_total_ is total energy (eV).

### 2.3. Antioxidant Activity of ***11a*** and ***11b***

Figure 3A shows the dose–responses curve of the CL responses obtained by the reaction of **11a** and **11b** with O_2_^−**.**^. Antioxidant activity was measured by the method described above. The results of IC_50_ (mean ± SD) are summarized in Figure 3B. O_2_^−**.**^ scavenging activity was stronger in compound **11b** than in **11a**. The IC_50_ value of **11b** was 8.5 (±1.5) µM and has higher O_2_^−**.**^ scavenging activity than **11a** (20.0 ± 5.0 µM), and inhibitory activity was higher in compounds **11a** and **11b** than in catechol and **8** alone. In particular, the IC_50_ of **11b** conjugated **7** was about 1/2.5 times that of **11a**. O_2_^−**.**^ scavenging activity was also about four times higher by **11a** than with **9** alone. On the other hand, the inhibitory activities of **12** and **13** were equal to or less than that of **7** (Figure 3C). They exhibited no effective O_2_^−**.**^ scavenging activity relative to **11a** and **11b**. However, compounds **12** and **13** had powerful antioxidant activities compared to **9** alone. 

Incubation in 10 mM phosphate buffer (pH 7.5) for 2 min at 25 °C; Responses were expressed as chemiluminescence for the concentration (−log[C], M) of antioxidants **11a** (**A**) and **11b** (**B**); All bars were expressed as the mean ± S.D. (**C**) **8**: *p*-Hydroquinone, **9**: catechol.

O_2_^−**.**^ scavenging activity was stronger in compounds **11a** and **11b** than in the natural compounds **1**, **3**, **4**, and **5**. The IC_50_ value of **11b** was almost equal to that of quercetin **5**. What are the chemical requirements that increase O_2_^−**.**^ scavenging activity? Differences in the spacers between **12**, **13** and **11a**, **b** affect inhibitory activity. In our previous study, we confirmed the relationship between the chemical hardness and antioxidant activity of antioxidants [3,5]. The chemical hardness concept can be applied to a comparison with the O_2_^−**.**^ scavenging power of antioxidants. By an MO calculation using B3LYP/6-31G(d), **11a** (χ = 3.565) and **11b** (χ = 3.480) had low absolute hardness and absolute electronegativity. Electron donation was higher in compounds **11a** and **11b**, with the χ values of **11a** and **11b** being smaller than the χ values of **2** (χ = 3.635), **3** (χ = 3.765), **4** (χ = 2.720), and **5** (χ = 3.825). Regarding the values for hardness, **11a** (η = 2.215) and **11b** (η = 2.340) were smaller than **1** (η = 2.650), **3** (η = 2.785), and **4** (η = 2.869), except for **2** (η = 1.835) and **5** (η = 1.836). The χ value of **11b** was smaller than that of **11a**. Although compounds **2** and **5** have a high driving force, their χ values were larger than those of **11a** and **11b**. These findings indicate that electron rich **11b** is more easily oxidized when scavenging O_2_^−**.**^ than **1**, **2**, **3**, **4**, **5**, and **11a**. 

### 2.4. Antioxidant Activity of the Metal Complexes **11a**–M^n+^

**Figure 4:**
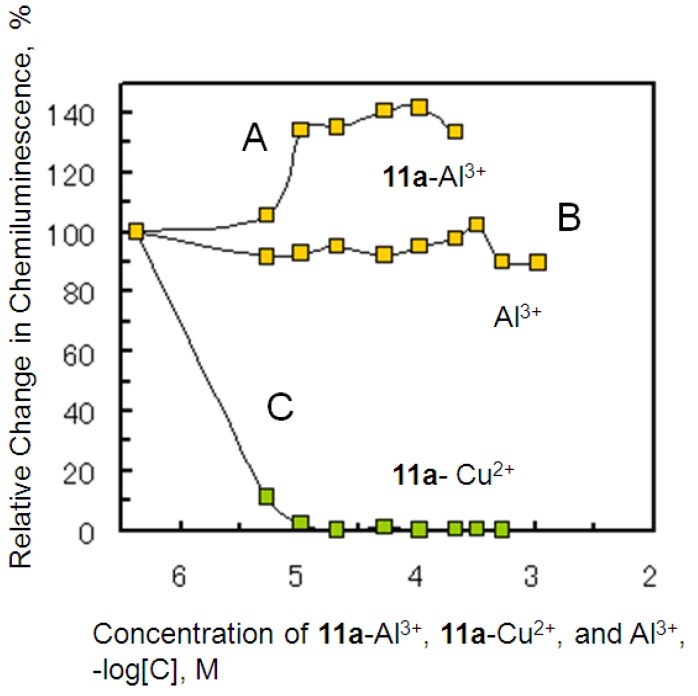
Dose–response curve of Al^3+^, **11a**–Al^3+^ and **11a**–Cu^2+^ complexes on O_2_^−**.**^ scavenging activity.

Compound **11a** forms 1:1 ratio solvated metal complexes with metal ions. To measure the O_2_^−**.**^ scavenging activity of the metal complexes **11a**–M^n+^, the molar ratio of **11a** to metal ions has to be 1:1 at any concentration. Therefore, we prepared the solvated **11a**–M^n+^ complexes of **11a** with the metal ions, Al^3+^, Cu^2+^, and La^3+^ ions, at several concentrations. Dose–chemical luminescence curves were obtained from the scavenging reaction of the **11a**–M^n+^ complexes under a constant concentration of O_2_**^−.^**, as shown in Figure 4. Although a decrease in CL was not clearly observed with the Al^3+^ (curve B in Figure 4), La^3+^ (data not shown) ions, and **11a**–Al^3+^ (curve A in Figure 4) with O_2_^−**.**^, a decrease in CL was seen in the scavenging reaction of the Cu^2+^ (data not shown) ion and **11a**–Cu^2+^ with O_2_^−**.**^ (curve C in Figure 4). From these results, it was shown that O_2_^−**.**^ scavenging activity increased in the following order: **11a**–Al^3+^ < **11a**–La^3+^ << **11a**–Cu^2+^ complexes, which indicates that Al^3+^, Cu^2+^, and La^3+^ ions were coordinated to the hydroxides of catechol rings since **11a** provides strong O_2_^−**.**^ scavenging activity, as shown in Figure 3. The complexes **11a**–Al^3+^ and **11a**–La^3+^ also had lower O_2_^−**.**^ scavenging activity. The sites of the scavenging reaction of **11a** were shown to be the hydroxides of the catechol rings. However, the **11a**–Cu^2+^ complex showed scavenging activity in spite of the coordination of **11a** with Cu^2+^ and the activity was higher than Cu^2+^ only at pH 7.4. In addition, we focused on the Al^3+^ ion, which has been implicated in Alzheimer’s disease through the process of amyloid β protein (Aβ) aggregation and oxidative free radicals [22]. The results shown in Figure 4A show that the Al^3+^ ion slightly accelerated the formation of O_2_^−**.**^ by interactions with **11a**. Al^3+^–**11a** complexes may have acted as a O_2_^−**.**^ generator.

**11a** and the Al^3+^ and Cu^2+^ ions were mixed at a molar ratio of 1:1; The values of the y-axis represent the relative changes in chemiluminescence as % inhibition.

**Figure 5:**
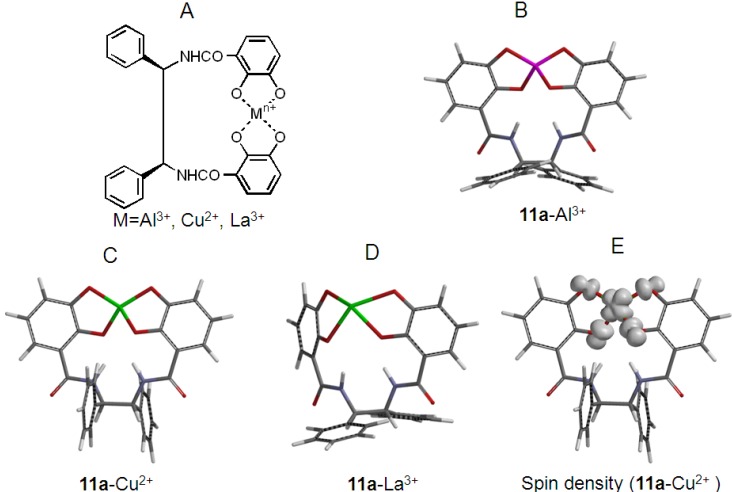
Most stable conformation of the **11a**–metal ion complexes after geometry optimization.

We prepared the solvated molecule complexes, **11a**–Al^3+^, **11a**–Cu^2+^, and **11a**–La^3+^, with a molar ratio of 1:1 (= [M^n+^]:[[**11a**]) according to the method of our previous study based on UV/Vis spectrophotometry [15,16]. Structures were obtained after geometry optimization using the B3LYP method at a molar ratio of 1:1 of M^n+^ and **11a**; the 6-31G(d) basis set was used for C, H, N, Al^3+^, and Cu^2+^, and the LANL2DZ basis set was used for optimization of the La^3+^ ion. Geometry optimized structures are shown in Figure 5. The atomic radii of Al^3+^ and Cu^2+^ ions were almost equal at 1.18 and 1.17 Å. Lanthanum (La) was 1.69 Å. These atomic radii were suitable to form the chelators of **11a** with Al^3+^, Cu^2+^, and La^3+^ ions. 

The chemical structure of the **11a**–metal ion complexes (**A**). Metal (M^n+^) = Al^3+^, Cu^2+^, and La^3+^ ions; Most stable conformation of the **11a**–M^n+^ complexes (**B**, **C**, and **D**): (i) M = Al^3+^; (ii) M = Cu^2+^; (iii) M = La^3+^; Spin density map of the **11a**–Cu^2+^ complex (**E**). At the B3LYP/6-31G(d) level.

**Figure 6:**
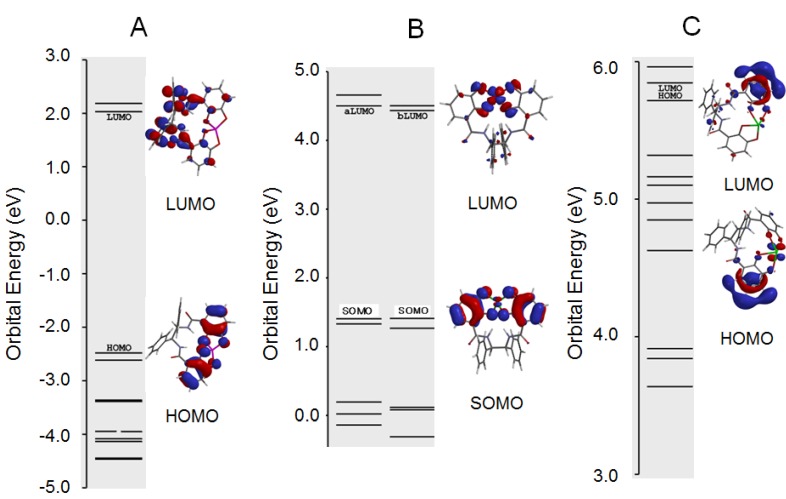
Orbital energy level and the HOMO, SOMO, and LUMO phases of the optimized **11a**–Metal complexes.

Why the **11a**–Al^3+^ complex did not have O_2_^−**.**^ scavenging activity was considered using molecular orbitals. Lebedev *et al.* reported that excess Ca^2+^ increased the rate of catechol oxidation with O_2_ [23]. However, Al^3+^ alone did not increase O_2_^−**.**^ levels in our study (Figure 4). Asano demonstrated that porphyrin in the porphyrin–Fe^III^ complex was difficult to exchange to porphyrin–Al^3+^ by the coordination with Al^3+^ [24,25]. In the HPX-XOD system, therefore, it is hard accepted that less O_2_^−**.**^ scavenging activity in the presence of Al^3+^ produced by Al–XOD, formed by the reaction of Al^3+^ with Fe–XOD. O_2_^−**.**^ produced by the HPX-XOD system is expected to interact with Al^3+^ in the center of the **11a**–Al^3+^ complex. To understand the mechanism by which Al^3+^ in the **11a**–Al^3+^ complex inhibits the O_2_^−**.**^ scavenging reaction, we theoretically analyzed an electronic energy diagram and molecular orbital of the geometry optimized **11a**–Al^3+^ complex. The calculated bond lengths of O–Al^3+^, O–Cu^2+^ and O–La^3+^ in the geometry optimized **11a**–Al^3+^, **11a**–Cu^2+^, and **11a**–La^3+^ complexes were 1.806, 2.332 and 2.349 Å, respectively, as displayed in Figure 5. O_2_^−**.**^ acted as an oxidant in the scavenging reaction with the **11a**–Al^3+^, **11a**–Cu^2+^, and **11a**–La^3+^ complexes, respectively (Figure 6).

Orbital energy (eV), ε_homo_, ε_lumo_, or ε_somo_ of **11a**–Al^3+^ (**A**), **11a**–Cu^2+^ (**B**), and **11a**–La^3+^ (**C**); At the B3LYP/6-31G(d) level for **11a**–Al^3+^ (ε_homo_= −2.48 and ε_lumo_ = 2.03) and UB3LYP/6-31G(d) level for **11a**–Cu^2+^ (ε_somo_ = 1.41 and ε_lumo_ = 4.50). At the B3LYP/lanL2DZ level for **11a**–La^3+^ (ε_homo_ = 5.78 and ε_lumo_ = 5.85).

In the scavenging reaction, O_2_^−**.**^ attacked the HOMO or SOMO of the metal-based MO in the **11a**–M^n+^ complexes. The SOMO distribution of Cu^2+^ ions in the **11a**–Cu^2+^ complex was more widely distributed than that of the Al^3+^ and La^3+^ ions in the less active **11a**–Al^3+^ and **11a**–La^3+^ complexes, as shown in Figure 7. Moreover, the SOMO energy (ε_somo_= +1.41 eV) of the **11a**–Cu^2+^ complex was significantly higher than that of the **11a**–Al^3+^ (ε_homo_= −2.48 eV) and **11a**–La^3+^ (ε_homo_= +5.72 eV) complexes. These results indicate the **11a**–Cu^2+^ complex was easily reduced to **11a**–Cu^+^. It is clear from this reason, therefore, that the value for the absolute hardness of Cu^2+^ was smaller than that for the Al^3+^ and La^3+^ ions since the coordinates **r**(χ, η) for the electronic structures of Al^3+^, Cu^2+^, and La^3+^ ions were **r**(70.48, 37.96), **r**(25.55, 4.7), and **r**(15.23, 14.43), respectively, in the ground state [15,16]. The SOMO of the *d* orbital of the Cu^2+^ ion in the **11a**–Cu^2+^ complex was more widely distributed in the center of the metal ion than that of the Al^3+^ and La^3+^ ions. The spin density of SOMO was presented in Figure 5E, where it was computed with B3LYP with 6-31G(d). 

## 3. Experimental

### 3.1. Materials and Methods

Pyrocatechol (2,3-dihydroxybenzoic acid), *p*-hydroquinone (2,5-dihydroxybenzoic acid), (1*R*,2*R*)-(+)-diphenylethylenediamine, 2-amino-2-hydroxylmethyl-1,3-propanediol (Tris), AlCl_3_, copper(II) chloride·2H_2_O (CuCl_2_·2H_2_O), and lanthanum chloride·7H_2_O (LaCl_3_·7H_2_O) were purchased from Wako Pure Chemical Industries Ltd. (Osaka, Japan). XOD (from buttermilk, Lot. D00098728) and hypoxanthine (HPX) were obtained from Calbiochem (La Jolla, CA, USA). 2-Methyl-6-*p*-methoxy- phenylethynylimidazo pyrazynone (MPEC) was purchased from Atto Corp. (Osaka, Japan). All experimental solutions were prepared with redistilled water. All other reagents were of the highest grade available. Compounds were detected on thin-layer chromatography (TLC) plates using iodine vapor or UV absorption. Silica gel column chromatography was performed on silica gel 60N (100 mesh, neutral; Kanto Chemical Co., Tokyo, Japan). UV/vis spectra were measured with a JASCO V-530 spectrophotometer (JASCO Corp., Tokyo, Japan). Chemiluminescence was measured with a Lumat LB9507 (Berthold Technologies, Bad Wildbad, Germany). Nuclear magnetic resonance (NMR) spectra were obtained with a Bruker AV300 or AV600 spectrometer (Bruker Corp, Karlsruhe, Germany) and NMR samples were dissolved in DMSO*d*_6_/CDCl_3_ (volume ratio = 5:2) with tetramethylsilane (TMS) as an internal reference. Fast atom bombardment mass (FAB) spectra were obtained on a JMS-HX110 spectrometer (JEOL Ltd., Tokyo, Japan), and relevant data were tabulated as *m/z*. Solvent systems were as follows, A: CHCl_3_–MeOH (20:1), and B: CHCl_3_–MeOH (10:1). 

### 3.2. Synthesis

Compounds **12** and **13** were prepared by the method described in our previous studies [11]. 2,3-Bis(benzyloxy)benzoic acid (**6**) (1.84 g, 5.5 mmol) in dry CHCl_3_ (25 mL) was added to CDI (1.13 g, 7.0 mmol) and stirred at room temperature for 1 h. To this solution, 0.56 g (2.6 mmol) of (1*R*,2*R*)-(+)-diphenylethylenediamine was added, and the resulting mixtures were stirred overnight. The solvent was removed in an evaporator, and the residue was taken up in CHCl_3_, washed with 5% NaHCO_3_, water, dried (Na_2_SO_4_), and filtered. The crude residues were chromatographed on silica gel (40 g) with CHCl_3_ as an eluent. **10a** was slightly soluble in MeOH and was recrystallized from hot MeOH. **10a** was obtained as a colorless solid in 43.0% yield (0.94 g); m.p. 85–86 °C (from hot MeOH). R_f_(A) = 0.86. High-resolution (HR)-FAB-MS (*p*-nitrobenzoate(NBA)) *m/z*: 845.3597 (Calcd. for C_56_H_49_O_6_N_2_: 845.3590 [M+H]^+^). 

Five % Pd-C (0.35 g) was added to a solution of **10a** (1.76 g, 2.08 mmol) in MeOH (200 mL). The mixture was shaken under a flow of H_2_. After the reaction was completed, the catalyst was removed with a glass filter (G3-4). The filtrate was suspended in aqueous MeOH and extracted several times with CHCl_3_. The organic layer was washed with 5% NaHCO_3_ and the aqueous layer was neutralized with 0.1 M HCl. The neutralized layers were extracted several times with CHCl_3_, washed with water, dried with Na_2_SO_4_, and filtered with a glass filter. The solvent was removed in the evaporator, and the solids were precipitated. The solids (180 mg) were dissolved in 1 mL MeOH and subjected to preparative thin layer chromatography (PTLC) using a 20 × 20 cm silica gel 60 F_254_ plate (2 mm thickness) (Merck Ltd., Whitehouse Station, NJ, USA) with chloroform/MeOH (20:1) as mobile phase. The disclosed bands of **11a** were then scraped off, dissolved in chloroform/methanol mixture (1:1) and filtered with absorbent cotton. The solvent was removed by evaporation to give **11a** as a yellowish brown powder. Yield 50% (90 mg). R_f_(B) = 0.57. m.p. 173–175 °C. ^1^H-NMR (DMSO-*d*_6_/CDCl_3_) δ: 5.58 (d, *J* = 4.1 Hz, 1H), 6.58 (t, *J* = 7.7 Hz, catecholic protons), 6.87 (d, *J* = 7.4 Hz, catecholic protons), 7.11–7.34 (m, 5H), 9.99 (broad, –NH–, 1H). ^13^C-NMR (DMSO-*d*_6_/CDCl_3_) ppm: 58.75, 115.1, 116.5, 117.6, 117.9, 127.9, 128.0, 128.7, 139.1, 146.7, 151.3, and 170.1. HR-FAB-MS (NBA) *m/z*: 485.1712 (Calcd. for C_28_H_25_O_6_N_2_: 485.1713 [M+H]^+^).

**10b**: prepared from **7** in a similar manner to that described for **10a**. Yield 48%. R_f_(A) 0.90. m.p. 106–107 °C (from hot MeOH). HR-FAB-MS (NBA) *m/z*: 845.3599 (Calcd. for C_56_H_49_O_6_N_2_: 845.3590 [M+H]^+^). 

**11b**: prepared from **10a** in a similar manner to that described for **11a**. Yield 55%. R_f_(B) 0.44. m.p. 170–171 °C. ^1^H-NMR (DMSO-*d*_6_/CDCl_3_) δ: 5.64 (d, *J* = 4.3 Hz, 1H), 6.68 (d, *J* = 8.8 Hz, *p*-hydroquinone protons), 6.87 (dd, *J* = 2.8 and 8.8 Hz, *p*-hydroquinone protons), 7.14–7.37 (m, 5H), and 9.34 (broad, –NH–, 1H). ^13^C-NMR (DMSO-*d*_6_/CDCl_3_) ppm: 57.09, 112.9, 114.9, 117.5, 121.3, 1126.8, 126.9, 127.7, 139.4, 148.9, 152.6, and 168.7. HR-FAB-MS (NBA) *m/z*: 485.1707 (Calcd. for C_28_H_25_O_6_N_2_: 485.1713 [M+H]^+^).

### 3.3. O_2_^−**.**^ Scavenging Assay

O_2_**^−.^** was generated using the HPX-XOD system. O_2_**^−.^** was subsequently emitted to reduce MPCE, which yielded a chemiluminescence product. The chemiluminescence concentration was measured as the chemiluminescence intensity (CL) with a lumicounter (Lumat LB9507, Berthold). XOD (0.1 U/mL) and HPX (0.75 mM) were prepared with 0.1 M phosphate buffer (pH 7.5). The O_2_**^−.^** scavenging reaction was performed in a total volume of 300 µL at 25 °C by mixing XOD (60 µL), 0.1 M phosphate buffer (final concentration 0.01 M phosphate buffer), MPEC (10 µL, 300 mM), and HPX (50 µL). Test compounds were mixed in nine test tubes (5 mL) just before adding HPX and the final concentrations of the test compounds, **11a** and **11b**, were 0.043, 0.53, 1.07, 2.13, 5.33, 10.66, 32.0, 53.3, and 106.6 × 10^−5^ M, respectively. The activities of compounds **1**, **2**, **3**, **4**, **5**, **12**, and **13** also were measured by a similar method.

A total of 0.01 M phosphate buffer was added to the reaction solution to a final volume of 3 mL and the solution was mixed. The reaction mixtures were then incubated at 25 °C in a water bath for 2 min. The reaction solution without test compounds was equilibrated to the desired level of CL output for 1 min. The half-maximal inhibitory concentration (IC_50_) was calculated from the dose–CL curve obtained from measured CL and the concentrations of the test compounds as shown in Figure 1 and Figure 4. 

### 3.4. O_2_**^−.^**Scavenging Assay of the **11a**–Metal Ion Complexes and Metal Ions

Solvated **11a**–metal ion complexes were prepared from the reaction of **11a** with Al^3+^, Cu^2+^, and La^3+^ ions, respectively, according to the UV/Vis titration method of previous studies [15,16]. Stock solutions of 3.2 mM of **11a** in EtOH and 2.89 mM of Al^3+^, Cu^2+^, and La^3+^ ions in 10 mM phosphate buffer (pH 7.5) were prepared, and **11a** and Al^3+^, Cu^2+^, and La^3+^ ions were mixed at a molar ratio of 1:1, respectively. According to the method of the O_2_**^−.^** Scavenging Assay, **11a**–metal ion (Al^3+^, Cu^2+^, and La^3+^) complexes were mixed with XOD (0.1 U/mL) in nine test tubes (5 mL) just before adding HPX and the final concentrations of the complexes were 0.038, 0.53, 1.06, 2.12, 5.30, 10.60, 21.2, 31.8, and 53.0 × 10^−5^ M, respectively. The mixtures were incubated at 25 °C for 2 min. Figure 5 shows the dose–CL curves of the **11a**–metal ions (Al^3+^ and Cu^2+^) complexes and Al^3+^ alone.

### 3.5. Computational Chemistry

Optimized conformations of the antioxidants and metal complexes in gas-phase were computed using the Spartan ‘10 (Wavefunction, Inc., Irvine, CA, USA) program. The lowest energy conformers determined with conformational search (Monte-Carlo method) computation at the MMFF94 level were optimized by the hybrid-density functional theory (DFT) using restricted B3LYP or unrestricted B3LYP functional with the split-valence 6-31G(d) basis set, and the results of the heavy atom **11a**–La^3+^ complex were obtained using B3LYP methods with Los Alamos effective core potentials (ECP) plus the double zeta (DZ) functions (LanL2DZ) basis set [26]. The B3LYP functional with the 6-31G(d) basis set was used on all other atoms. The geometries and electronic energy of the **11a**–Cu^2+^ complex with a spin multiplicity (S) of S = 2 were computed using unrestricted B3LYP functional with the 6-31G(d) basis set. The zero-point and thermal corrections of **11a** and **11b** were obtained by a complete vibrational analysis using single points on the 6-31G(d) optimized structures. 

## 4. Conclusions

We have demonstrated the synthesis and O_2_**^−.^** scavenging activity of some new potent antioxidants, **11a** and **11b**, conjugated with catechol and *p*-hydroquinone, and the solvated complexes, **11a**–Al^3+^ and **11a**–La^3+^, of **11a** with the Al^3+^ and La^3+^ ions exhibited no scavenging activity; however, the **11a**–Cu^2+^ complex provided strong superoxide scavenging activity, which was more powerful than the Cu^2+^ ion alone. This was attributed to the spin multiplicity of the **11a**–Cu^2+^ complex being doublet and it having an ε_SOMO_ of +1.41 eV. The SOMO of the **11a**–Cu^2+^ complex was expanded in the center of the Cu^2+^ ion. The SOMO plays an important role in the superoxide scavenging activity of the **11a**–Cu^2+^ complex. The **11a**–Cu^2+^ complex increased the chemical softness and decreased the absolute hardness. The electronic state of **11a** contributed to the increase in chemical softness. 

Although **11a**–Al^3+^ had a small χ value, its η value was 2.255, which was smaller than that of **11a**–Cu^2+^. This finding indicates that it is difficult to act on as the driving force of the redox reaction. The HOMO distribution of the **11a**–Al^3+^ and **11a**–La^3+^ complexes was not in the metal center. In addition, the slight increase inO_2_**^−.^** by the **11a**–Al^3+^complex indicated the important role of Al^3+^ in accelerating the formation of O_2_**^−.^**, which ultimately contributes to Alzheimer’s disease through the process of oxidative free radicals. We showed the importance of the design of molecular and metal complexes using chemical hardness to control the antioxidant activity of O_2_**^−.^**, and developed the powerful antioxidants, **11a**, **11b**, **12**, **13** and the solvated complex **11a**–Cu^2+^ system. 
